# Comprehensive analysis of risk factors and metabolic profiling in preclinical atherosclerosis: a cross-sectional study

**DOI:** 10.3389/fphys.2025.1677194

**Published:** 2025-10-30

**Authors:** Li Liu, Fengrong Wang, Lijie Jiang, Tiehong Liu, Linlin Dong, Tianjiao Zhang, Guoling Hu

**Affiliations:** ^1^ Department of Geriatrics, Affiliated Zhongshan Hospital of Dalian University, Dalian, Liaoning, China; ^2^ Department of Cardiology, Affiliated Hospital of Liaoning University of Traditional Chinese Medicine, Shenyang, Liaoning, China

**Keywords:** preclinical, atherosclerosis, risk factors, metabolomics, mass spectrometry

## Abstract

**Aim:**

This study aimed to explore the factors influencing preclinical atherosclerosis (PCA) and provide evidence-based recommendations for its prevention. Non-targeted metabolomics technology was utilized to identify potential metabolic biomarkers associated with PCA.

**Materials and Methods:**

Data on general conditions, risk factors, and metabolic biochemical test results were collected from both the PCA group patients and the control group people. Blood plasma metabolites were analyzed using LC-MS/MS, which is a powerful technique that couples the separation power of liquid chromatography (LC) with the highly sensitive and specific detection of tandem mass spectrometry (MS/MS), making it indispensable for the comprehensive and accurate metabolic profiling required in preclinical atherosclerosis studies. Metabolites were annotated using the HMDB and LIPIDMaps databases, and differential metabolite pathways were enriched using the KEGG database.

**Results:**

Significant differences were observed between the two groups in terms of BMI, diet habits, smoking, physical activity, hypertension, and diabetes. Multivariate analysis identified smoking, high-salt diet, hypertension, and diabetes as significant risk factors for PCA. Biochemical blood tests revealed significantly elevated levels of triglycerides, LDL-C, GLU, and UA in the PCA group compared to the control group. Metabolomic analysis identified 105 differential metabolites in positive ion mode (29 upregulated and 76 downregulated) and 105 differential metabolites in negative ion mode (39 upregulated and 66 downregulated). The primary metabolic differences between the groups were related to lipid metabolism, inflammation-mediated processes, and amino acid metabolism.

**Conclusion:**

The incidence of PCA is influenced by smoking, unhealthy diet habits, hypertension, and diabetes. PCA patients frequently exhibit abnormalities in lipid metabolism, glucose metabolism, and purine metabolism. Metabolomic studies indicate that the metabolic differences in PCA primarily involve lipid metabolism, energy metabolism, and amino acid metabolism.

## 1 Introduction

Preclinical atherosclerosis (PCA) is characterized by the presence of atherosclerosis (AS) plaques without evident clinical symptoms. The progression of AS plaques, which can lead to cardiovascular events, typically occurs over an extended period and is influenced by various risk factors (Kawai et al., 2024). Currently, the assessment of cardiovascular risk relies on evaluating a patient’s risk factors to guide primary prevention strategies. However, approximately 20% of patients experience their first or recurrent acute myocardial infarction without prior warning signs (SCORE2 working group and ESC Cardiovascular risk collaboration, 2021; Global et al., 2023; Gulinuer and Nuerguli, 2023). Autopsy studies have further revealed that 55% of myocardial infarction cases involve AS plaques causing less than 50% arterial stenosis, suggesting that many of these patients may have had PCA prior to the onset of clinical disease (Li et al., 2016). Consequently, early screening for PCA and the implementation of effective interventions are critical to prevent progression to advanced or terminal stages of cardiovascular disease. Metabolomics focuses on the systematic analysis of endogenous metabolites within an organism, organ, or system, as well as those influenced by external environmental factors, offers a promising approach for studying early-stage AS. Despite its potential, research in this area remains limited. This study utilized liquid chromatography-tandem mass spectrometry (LC-MS/MS) to analyze and compare plasma metabolite profiles between PCA group and control group, aiming to identify differential biomarkers associated with PCA and elucidate potential metabolic pathways involved in its pathogenesis. This study complies with national clinical trial regulations and has been reported in line with the STROCSS criteria (Agha et al., 2025).

## 2 Materials and methods

### 2.1 Patient recruitment and clinical data collection

In this study, a total of 210 patients meeting the diagnostic criteria for PCA were recruited from Affiliated Zhongshan Hospital of Dalian University between July 2023 and February 2024. Additionally, 50 healthy volunteers who underwent routine physical examinations at the hospital’s Health Examination Center during the same period were included as the control group. The study protocol was reviewed and approved by the Ethics Committee of Affiliated Zhongshan Hospital of Dalian University (Approval No. KY2023-096-1).

### 2.2 Diagnostic criteria of PCA

The carotid intima-media thickness (IMT) of patients was assessed using the Hitachi LOGIQ color Doppler ultrasound diagnostic instrument by an experienced ultrasound physician. The diagnostic criteria for PCA were defined as IMT ≥0.9 mm or the presence of AS plaques, either single or multiple, with a thickness >1.2 mm protruding from the intima surface. Plaques observed at each site were repeatedly scanned, and the location of the largest plaque was recorded, with its maximum length and thickness measured (Gianaros et al., 2022; Caughey et al., 2018; Touboul et al., 2012; Mfeukeu-Kuate et al., 2022).

The inclusion criteria for the PCA group: (1) Meeting the diagnostic criteria for PCA. (2) Aged 40–75 years, with no gender restrictions. (3) Informed and willing to participate in the scale assessments. Inclusion criteria for the control group: (1) Healthy individuals who do not meet any of the diagnostic criteria for PCA. (2) Aged 40–75 years, with no gender restrictions; (3) Willing to participate in the study and having provided written informed consent.

The exclusion criteria: (1) Unwillingness to cooperate with the study, or presence of impaired consciousness, psychiatric disorders, or other conditions that may hinder proper participation. (2) History of cerebrovascular diseases (e.g., stroke, transient ischemic attack). (3) Presence of peripheral vascular disease due to any other etiology. (4) Comorbid coronary artery disease or cardiac dysfunction. (5) Comorbid familial hypercholesterolemia, connective tissue disorders, or vasculitis. (6) Severe systemic diseases affecting major organs (e.g., heart, lung, liver, or kidney), malignancy, poorly controlled diabetes mellitus, or refractory hypertension. (7) Acute infectious diseases or systemic stress due to other underlying conditions.

### 2.3 Investigation of influencing factors and detection of biochemical indicators

In this study, the General Situation Questionnaire for PCA was developed based on a comprehensive review of preclinical literature, clinical investigations, and expert consultations. Participants were surveyed to collect general information, potential risk factors, and biochemical test results, including triglycerides (TG), total cholesterol (TC), low-density lipoprotein cholesterol (LDL-C), high-density lipoprotein cholesterol (HDL-C), non-HDL cholesterol, apolipoprotein A1 (APOA1), apolipoprotein B (APOB), the APOA1/APOB ratio, glucose (GLU), and uric acid (UA).

### 2.4 Non-targeted metabolomics studies

#### 2.4.1 Extraction of metabolite from blood plasma samples

Blood plasma samples collection: On the morning of day 1 after enrollment, fasting venous blood was drawn from the antecubital vein of all subjects into vacuum anticoagulant blood collection tubes (K3EDTA). The samples were centrifuged at 4 C and 3000 rpm/min for 10 min. The supernatant was then collected, aliquoted at 0.2 mL per tube into 2 mL cryovials, and properly labeled. The aliquots were rapidly frozen in liquid nitrogen for 15 min and stored in a −80 C freezer for future use.

The plasma samples (100 μL) were placed in EP tubes and resuspended with pre-chilled 80% methanol (400 μL)using thorough vortexing. The samples were then incubated on ice for 5 min and centrifuged at 15,000 × g and 4 C for 20 min. A portion of the supernatant was diluted to a final concentration of 53% methanol using LC-MS grade water. The samples were subsequently transferred to a fresh Eppendorf tube and centrifuged again at 15,000 × g and 4 C for 20 min. Finally, the supernatant was injected into the LC-MS/MS system for analysis (Xue et al., 2022; Xiao et al., 2021).

#### 2.4.2 UHPLC-MS/MS analysis

Ultra-High-Performance Liquid Chromatography-Tandem Mass Spectrometry (UHPLC-MS/MS) offers superior chromatographic resolution, speed, and sensitivity compared to conventional LC-MS/MS, enabling more precise separation and identification of complex metabolite mixtures. UHPLC-MS/MS analysis were conducted using a Vanquish UHPLC system (Thermo Fisher, Germany) coupled with either an Orbitrap Q Exactive™ HF mass spectrometer or an Orbitrap Q Exactive™ HF-X mass spectrometer (Thermo Fisher, Germany). Samples were injected onto a Hypersil Gold column (100 × 2.1 mm, 1.9 μm) with a 12-min linear gradient at a flow rate of 0.2 mL/min. The eluents for positive and negative polarity modes were eluent A (0.1% formic acid in water) and eluent B (methanol). The solvent gradient was programmed as follows: 2% B, 1.5 min; 2%–85% B, 3 min; 85%–100% B, 10 min; 100%–2% B, 10.1 min; and 2% B, 12 min. The Q Exactive™ HF mass spectrometer was operated in positive/negative polarity mode with the following parameters: spray voltage of 3.5 kV, capillary temperature of 320 °C, sheath gas flow rate of 35 psi, auxiliary gas flow rate of 10 L/min, S-lens RF level of 60, and auxiliary gas heater temperature of 350 C.

The mass range for both MS1 and MS2 was set to m/z 100–1000; MS1 scan resolution: 70,000, MS2 scan resolution: 17,500; Higher-energy collisional dissociation (HCD) with collision energies of 20%, 40%, and 60% (Stepped NCE).

#### 2.4.3 Data processing and metabolite identification

The raw data files obtained from UHPLC-MS/MS were processed using Compound Discoverer 3.3 (CD3.3, ThermoFisher) to perform peak alignment, peak picking, and metabolite quantitation. Key parameters were configured as follows: peak areas were corrected using the first quality control (QC) sample, with a mass tolerance of 5 ppm, a signal intensity tolerance of 30%, and a minimum intensity threshold. Subsequently, peak intensities were normalized to the total spectral intensity. The normalized data were utilized to predict molecular formulas based on additive ions, molecular ion peaks, and fragment ions. These peaks were then matched against the mzCloud (https://www.mzcloud.org/), mzVault, and MassList databases to obtain accurate qualitative and relative quantitative results. Statistical analyses were conducted using R (version 3.4.3), Python (version 2.7.6), and CentOS (release 6.6). For non-normally distributed data, standardization was performed using the formula: sample raw quantitation value/(sum of sample metabolite quantitation values/sum of QC1 metabolite quantitation values) to derive relative peak areas. Compounds with coefficient of variation (CV) values exceeding 30% in QC samples were excluded, yielding the final metabolite identification and relative quantification results.

### 2.5 Statistical analysis of data

In this study, statistical analysis was conducted using SPSS 27.0 software. Categorical data were described using frequencies and percentages, and group comparisons were performed using the Pearson *χ* ^*2*^ test. Continuous data were expressed as mean ± standard deviation (mean ± SD), and comparisons between two groups were conducted using independent samples *t*-tests. Variables with *P* < 0.05 in univariate analysis were selected for further multivariate analysis using binary logistic regression to identify influencing factors of PCA. *P* < 0.05 was considered statistically significant, while *P* < 0.01 was deemed highly statistically significant.

The metabolites were annotated using the KEGG database (https://www.genome. jp/kegg/pathway.html), HMDB database (https://hmdb.ca/metabolites), and LIPIDMAPS database (http://www.lipidmaps.org/). Principal component analysis and partial least squares discriminant analysis (PLS-DA) were conducted using metaX, a flexible and comprehensive software for processing metabolomics data (Sun et al., 2025). Univariate analysis (*t*-test) was applied to calculate the statistical significance (*P*-value). Metabolites with a variable importance in projection (VIP) > 1, *P* < 0.05, and fold change (FC) ≥ 2 or ≤0.5 were identified as differential metabolites. Volcano plots, generated using the ggplot2 package in R, were employed to visualize metabolites of interest based on log2(Fold Change) and -log10(P-value). For clustering heatmaps, the intensity areas of differential metabolites were normalized using z-scores and visualized using the Pheatmap package in R. The correlation between differential metabolites was analyzed using the cor () function in R (method = Pearson), and the statistical significance of these correlations was calculated using the cor.mtest () function in R. *P* < 0.05 was considered statistically significant, and correlation plots were generated using the corrplot package in R. The functional roles of these metabolites and their associated metabolic pathways were investigated using the KEGG database. Metabolic pathway enrichment analysis was performed, with pathways considered enriched if the ratio satisfied x/n > y/N and statistically significant if the *P* < 0.05.

## 3 Results

### 3.1 General data analysis

#### 3.1.1 Age and sex

In this study, the PCA group consisted of 210 patients, including 116 males (55.23%) and 94 females (44.76%). The control group consisted of 50 individuals, including 23 males (46.0%) and 27 females (54.0%). The age range of both groups was 40–75 years, with a mean age of 63.60 ± 7.30 years of the PCA group and 62.66 ± 7.73 years of the control group. No statistically significant differences were observed between the two groups in terms of gender (*P* > 0.05) or age *(P* > 0.05), as detailed in Table 1.

**TABLE 1:** Age and sex of research objects.

Gender/Age	PCA group (n = 210)	Control group (n = 50)	*χ* ^2^ value/*t* value	*P*
Gender			1.385	0.239
Male	116	23		
Female	94	27		
Age	63.60 ± 7.30	62.66 ± 7.73	0.809	0.419

#### 3.1.2 Univariate analysis of influencing factors

A comparison of influencing factors between PCA group and control group revealed that the prevalence of smoking, hypertension, and diabetes were significantly higher in PCA group than control group (*P* < 0.05). Furthermore, a significantly lower proportion of individuals in PCA group reported regular exercise habits compared to control group (*P* < 0.05), while PCA group exhibited a significantly higher BMI level than the control group *(P* < 0.05). Statistically significant differences were also observed in dietary habits between the two groups (*P* < 0.05). However, no significant differences were found between the groups regarding education level, occupation, drinking habits, or family history of cardiovascular and cerebrovascular diseases (*P* > 0.05), as detailed in Table 2.

**TABLE 2:** Univariate analysis of influencing factors of research objects.

Influencing factors	PCA group (n = 210)	Control group (n = 50)	*χ* ^2^ value/*t* value	*P*
Education level			4.634	0.099
Primary School	31 (14.76)	3 (6.00)		
Secondary School	164 (78.10)	40 (80.00)		
University	15 (7.14)	7 (14.00)		
Occupation			2.421	0.490
Farmer	19 (9.05)	3 (6.00)		
Workers	51 (24.29)	8 (16.00)		
Intellectuals	126 (60.00)	35 (70.00)		
Others	14 (6.67)	4 (8.00)		
Eating habits			9.931	0.019
Light	33 (15.71)	12 (24.00)		
General	71 (33.81)	25 (50.00)		
Greasy	49 (23.33)	5 (10.00)		
High salt	57 (27.14)	8 (16.00)		
Drinking habits			0.621	0.431
Yes	25 (11.90)	4 (8.00)		
No	185 (88.10)	46 (92.00)		
Smoking habit			6.247	0.012
Yes	154 (73.33)	45 (90.00)		
No	56 (26.67)	5 (10.00)		
Exercise (≥3 times/week, duration ≥30 min)			6.572	0.010
Yes	130 (61.90)	21 (42.00)		
No	80 (38.10)	29 (58.00)		
Suffered from hypertension			30.274	<0.001
Yes	140 (66.67)	12 (24.00)		
No	70 (33.33)	38 (76.00)		
Suffered from diabetes			4.712	0.030
Yes	171 (81.43)	47 (94.00)		
No	39 (18.57)	3 (6.00)		
Family history of cardio-cerebrovascular disease			0.651	0.420
Yes	117 (55.71)	31 (62.00)		
No	93 (44.29)	19 (38.00)		
BMI	24.42 ± 2.31	23.62 ± 1.97	2.277	0.024

#### 3.1.3 Multivariate analysis of influencing factors

Based on the results of the univariate analysis, factors exhibiting statistically significant differences between the two groups were included in the multivariate analysis. The results indicated that smoking, a high-salt diet, hypertension, and diabetes were identified as significant risk factors for PCA (*P* < 0.05), while no significant associations were observed for other factors (*P* > 0.05). Specifically, smoking, a high-salt diet, hypertension, and diabetes were all associated with increased odds ratios (*OR* > 1). Smokers had a 4.296-fold higher risk of developing PCA compared to non-smokers (*OR* = 4.296). Individuals with a high-salt diet exhibited a 2.839-fold higher risk compared to those without this dietary habit (*OR* = 2.839). Hypertension was associated with a 7.337-fold higher risk compared to individuals without hypertension (*OR* = 7.337). Similarly, patients with diabetes had a 4.320-fold higher risk compared to those without diabetes (*OR* = 4.320), as detailed in Table 3.

**TABLE 3:** Logistic correlation analysis of influencing factors.

Influencing factors	*B*	*SE*	*Wald*	*P*	*Exp(B)*	*95% CI*
BMI	0.053	0.085	0.380	0.537	1.054	0.892∼1.245
Ordinary diet			7.108	0.069		
Greasy diet	0.692	0.562	1.518	0.218	1.997	0.664∼6.003
High salt diet	1.043	0.507	4.238	0.040	2.839	1.051∼7.667
Light diet	−0.218	0.656	0.111	0.739	0.804	0.222∼2.908
Smoking habit	1.458	0.561	6.750	0.009	4.296	1.430∼12.904
Exercise (≥3 times/week Time for 30 min or more)	−0.509	0.366	1.935	0.164	0.601	0.293∼1.232
Suffered from hypertension	1.993	0.391	26.011	<0.001	7.337	3.411∼15.780
Suffered from diabetes	1.463	0.680	4.628	0.031	4.320	1.139∼16.384

#### 3.1.4 Biochemical tests related to metabolism in plasma

Biochemical markers related to metabolism in plasma, including TG, TC, HDL-C, LDL-C, non-HDL, APOA1, APOB, APOA1/APOB ratio, GLU, and UA levels, were compared between PCA group and control group. Compared to control group, PCA group exhibited significantly higher levels of TG, LDL-C, GLU, and UA (*P* < 0.05). In contrast, HDL-C levels, APOA1 levels, and the APOA1/APOB ratio were significantly lower in PCA group (*P* < 0.05). No significant differences were observed between the two groups in terms of TC levels, non-HDL, or APOB (*P* > 0.05), as detailed in Table 4.

**TABLE 4:** Univariate analysis of metabolism-related biochemical markers in plasma.

Metabolism-related biochemical markers	PCA group (n = 210)	Control roup (n = 50)	*t* value	*P*
TG (mmol/L)	1.74 ± 0.79	1.48 ± 0.47	3.067	0.003
TC (mmol/L)	4.84 ± 1.10	4.66 ± 0.64	1.510	0.134
HDL-C (mmol/L)	1.14 ± 0.24	2.83 ± 0.54	−21.478	<0.001
LDL-C (mmol/L)	3.03 ± 0.86	1.21 ± 0.28	25.487	<0.001
non-HDL (mmol/L)	3.71 ± 1.09	3.53 ± 0.76	1.317	0.191
APOA1 (g/L)	0.99 ± 0.18	1.06 ± 0.20	−2.441	0.015
APOB(g/L)	0.94 ± 0.25	0.88 ± 0.17	1.901	0.060
APOA1/APOB	1.13 ± 0.38	1.25 ± 0.32	−2.019	0.045
GLU (mmol/L)	5.88 ± 1.52	5.30 ± 0.84	3.653	<0.001
UA (μmol/L)	346.77 ± 90.51	314.10 ± 72.66	2.375	0.018

### 3.2 Non-targeted metabolome

#### 3.2.1 PLS-DA analysis between groups

**FIGURE 1:**
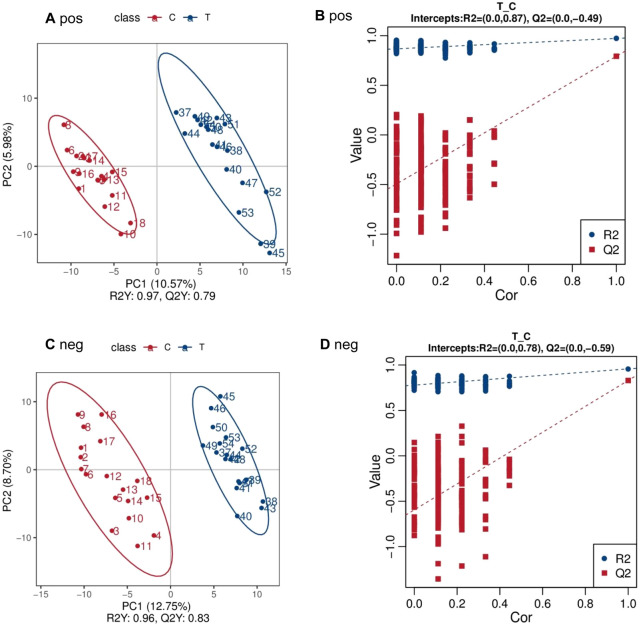
PLS-DA scatter point diagram and sequencing test diagram of positive and negative ion mode between PCA group (T) and control group (C) **(A)** pos **(B)** pos **(C)** neg **(D)** neg.

The PLS-DA score plot and permutation test plot were generated to compare PCA group (T) and control group (C), as illustrated in Figure 1. Significant differences in metabolite profiles between PCA group and control group were identified in both positive and negative ion modes (Figures 1A,C). The permutation test results (Figures 1B,D) confirm the robustness and reliability of the PLS-DA model.

#### 3.2.2 Differential metabolites between groups

**FIGURE 2:**
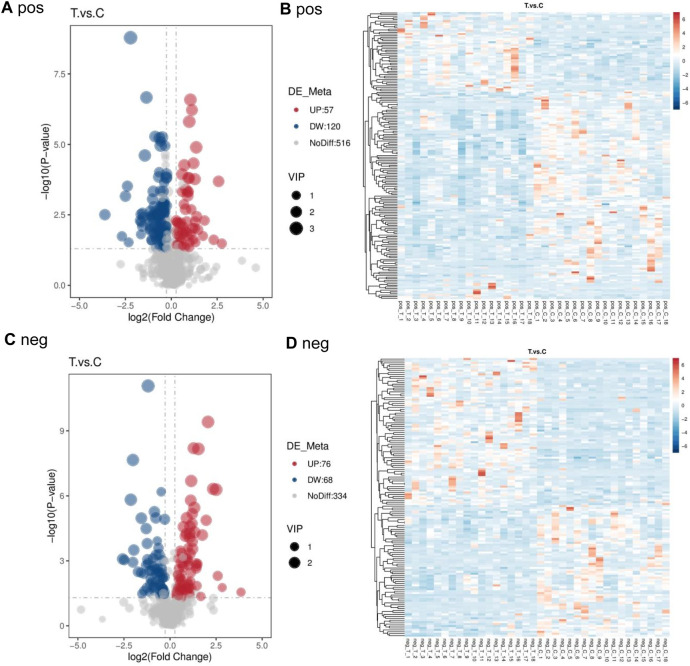
Volcano map and cluster heat map of two groups in positive and negative ion mode **(A)** pos **(B)** pos **(C)** neg **(D)** neg.

Compared to the control group, 177 differential metabolites were identified in PCA group under positive ion mode, with VIP >1.0, FC > 1.2 or FC < 0.833, and P-value <0.05. Among these, 57 metabolites were upregulated, and 120 were downregulated. Under negative ion mode, 144 differential metabolites were identified, with 76 upregulated and 68 downregulated. Table 5 lists the top 30 differential metabolites for both ion modes. The volcano plots in Figures 2A,C illustrate the distribution of these metabolites in positive and negative ion modes, respectively, while the corresponding clustering heatmaps are presented in Figures 2B,D.

**TABLE 5:** Differences in metabolites between PCA group and control group in positive and negative ion mode (top 30).

Compound_ID	Name	Molecular formula	MW	Annotation method	Level of annotation	RT [min]	m/z	FC	Pvalue	VIP	Up/Down
Com_18897_neg	dUDP	C9H14N2O11P2	388.01	HMDB0001000; KEGG C01346 LMST02030026	Level 3	1.82	387	0.44	8.47e-12	3.13	down
Com_5126_neg	PE 0–16:0_20:4	C41H76NO7P	725.53	--	Level 2	9.16	724.53	4.18	3.88e-10	2.82	up
Com_7737_pos	2-(3,4-dimethoxyphenyl) quinoline	C17H15NO2	265.11	--	Level 2	7.17	266.12	0.21	1.65e-09	2.98	down
Com_12159_neg	PE-Cer 12:1; 20/22:0	C36H73N2O6P	660.52	--	Level 2	9.47	659.51	2.43	6.31e-09	2.46	up
Com_3264_neg	N-lactoyl-phenylalanine	C12H15NO4	237.10	HMDB0062175	Level 3	5.65	236.09	2.91	6.81e-09	2.82	up
Com_15245_neg	LPE 0–22:2	C27H54NO6P	519.37	--	Level 2	11.19	518.36	0.25	2.21e-08	2.68	down
Com_4909_neg	Acetylcarnitine	C9H17NO4	203.12	HMDB0240773; KEGG C02571 LMFA07070060	Level 3	5.65	202.11	2.22	2.05e-07	2.65	up
Com_18995_pos	Dl-Lanthionine	C6H12N2O4S	208.05	HMDB0251521	Level 3	5.5	209.06	0.39	2.16e-07	2.43	down
Com_15018_pos	3-hydroxy-1,5-diphenylpentan-1-one	C17H18O2	276.11	--	Level 2	5.58	277.12	2.06	2.61e-07	2.46	up
Com_10154_neg	PC 14:0_16:0	C38H76NO8P	751.54	LMGP01010560	Level 2	10.66	750.53	5.05	4.79e-07	2.59	up
Com_681_neg	PC 16:0_18:1	C42H82NO8P	805.58	LMGP01012146	Level 2	11.59	804.57	5.67	5.15e-07	2.84	up
Com_2675_pos	PC 16:1_16:1	C40H76NO8P	1459.05	--	Level 2	11.01	730.53	2.18	6.01e-07	2.05	up
Com_18680_neg	PC 20:1_18:2	C46H86NO8P	857.62	--	Level 2	11.16	856.61	0.72	6.51e-07	1.26	down
Com_16448_neg	Cer 29:0; 20/12:0; 0 (FA 18:0)	C59H117NO5	919.89	--	Level 2	11.02	918.88	0.23	1.51e-06	2.7	down
Com_16493_pos	Phosphopyruvic acid	C3H5O6P	167.98	KEGG C00074	Level 3	9.53	168.99	1.97	1.56e-06	2.48	up
Com_22636_neg	LNAPE 18:2/N-17:0	C40H76NO8P	729.53	--	Level 2	10.7	728.52	2.18	1.61e-06	2.32	up
Com_5693_neg	PC 0–17:0_14:0	C39H80NO7P	751.58	--	Level 2	10.72	750.57	2.49	3.53e-06	2.54	up
Com_19779_pos	Gentisic acid	C7H6O4	154.03	HMDB0000152; KEGG C00628	Level 3	5.43	347.01	0.54	5.25e-06	2	down
Com_864_neg	L-(−)-Glyceric acid	C3H6O4	106.03	--	Level 3	1.36	105.02	0.56	5.41e-06	2.17	down
Com_341_pos	LPC 0–16:0	C24H52NO6P	481.35	LMGP01060010	Level 2	9.95	482.36	0.71	5.61e-06	2.2	down
Com_9_pos	1-Stearoylglycerol	C21H42O4	358.31	HMDB0244009	Level 2	10.49	341.3	0.64	5.94e-06	2.07	down
Com_23022_neg	N-Acetylneuraminic acid	C11H19NO9	309.11	HMDB0000230; KEGG C00270	Level 3	6.13	308.1	2.32	6.67e-06	2.18	up
Com_492_pos	Nonadecanoic acid	C19H38O2	298.29	KEGG C16535; LMFA01010019	Level 3	10.49	299.29	0.67	7.89e-06	1.48	down
Com_5766_neg	LPC 0–18:2	C26H52NO6P	551.36	--	Level 2	9.92	550.35	0.59	1.05e-05	2.08	down
Com_10396_neg	LPI 20:4	C29H49O12P	620.30	LMGP06050006	Level 2	8.47	619.29	1.88	1.06e-05	2.49	up
Com_17366_neg	PC 0–18:0_22:6	C48H86NO7P	865.62	--	Level 2	9.54	864.61	0.34	1.07e-05	2.29	down
Com_236_pos	LPC 0–16:1	C24H50NO6P	479.34	LMGP01060031	Level 2	9.75	480.34	0.67	1.11e-05	2.24	down
Com_6861_pos	5-hydroxy-4-methoxy-5,6-dihydro-2H-pyran-2-one	C6H8O4	144.04	--	Level 2	1.21	145.05	0.79	1.12e-05	1.53	down
Com_506_neg	N1-[4-(2-thienylthio)phenyl]-4-chlorobenzamide	C17H12ClNOS2	345.00	--	Level 2	11.8	343.99	0.82	1.21e-05	1.92	down
Com_17822_pos	4-(2,3-dihydro-1,4-benzodioxin-6-yl)butanoic acid	C12H14O4	100.05	--	Level 2	1.3	223.09	2.56	1.27e-05	2.4	up

Annotation method: HMDB (Human Metabolome Database), KEGG (Kyoto Encyclopedia of Genes and Genomes), and LMGP/LMST/LMFA (LIPID, MAPS, classification for lipids). Entries listed as “--“ indicate that the annotation was based on spectral interpretation and/or an in-house library without a match to the specified public databases. Level of annotation: Confidence levels are assigned according to the Metabolomics Standards Initiative (MSI) guidelines. Level 1: Confidently identified by comparison with an authentic standard. Level 2: Putatively annotated based on spectral similarity to a library or characteristic diagnostic evidence (e.g., for a lipid class). Level 3: Putatively characterized compound class based on precise mass and/or spectral data.

**FIGURE 3:**
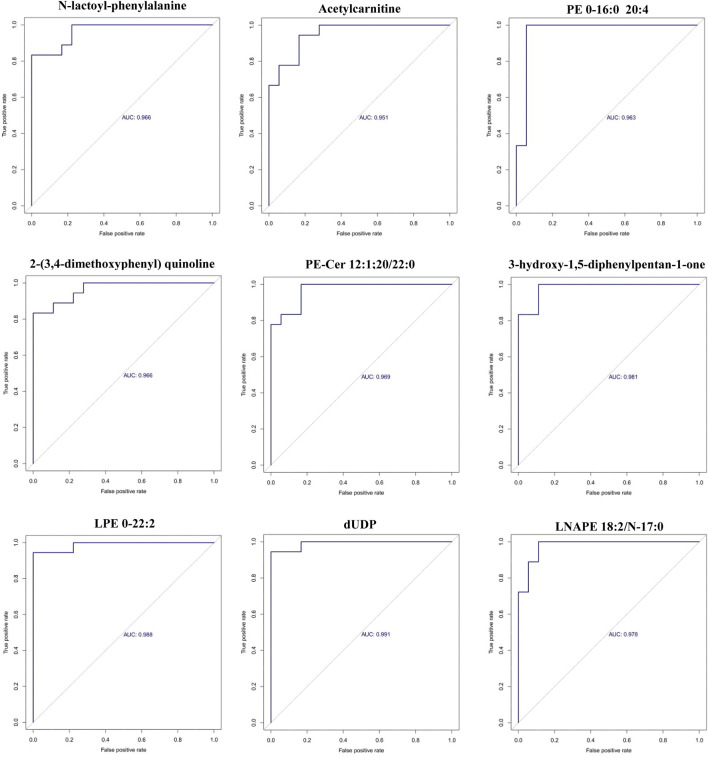
ROC curves of the 9 differential metabolites between PCA group and control group (x-axis: False positive rate; y-axis: True positive rate).

Receiver operating characteristic (ROC) curve analysis was conducted on the top 30 screened differential metabolites. The diagnostic value of each metabolite for PCA was assessed by calculating the area under the curve (AUC). The results showed that the AUC values for all metabolites ranged from 0.80 to 1.0, and 9 differential metabolites had an AUC greater than 0.95, as presented in Figure 3.

#### 3.2.3 Enrichment analysis of KEGG pathways of differential metabolites

**FIGURE 4:**
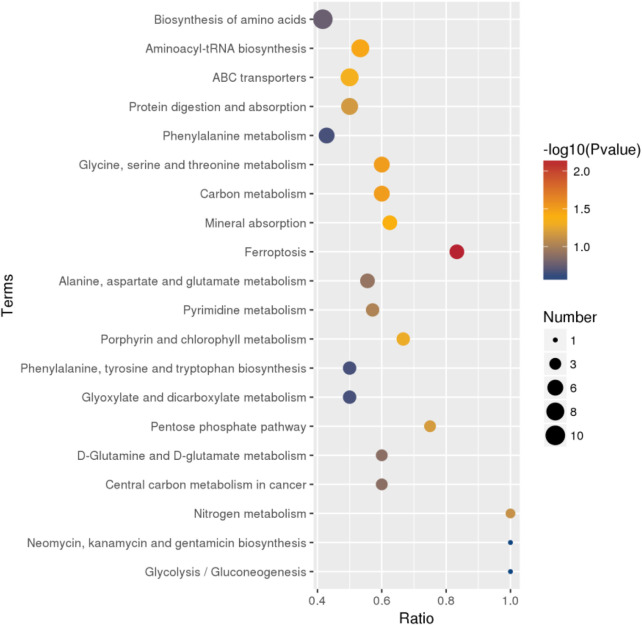
Bubble map of KEGG enrichment analysis of differential metabolites between PCA group and control group (top 20).

**FIGURE 5:**
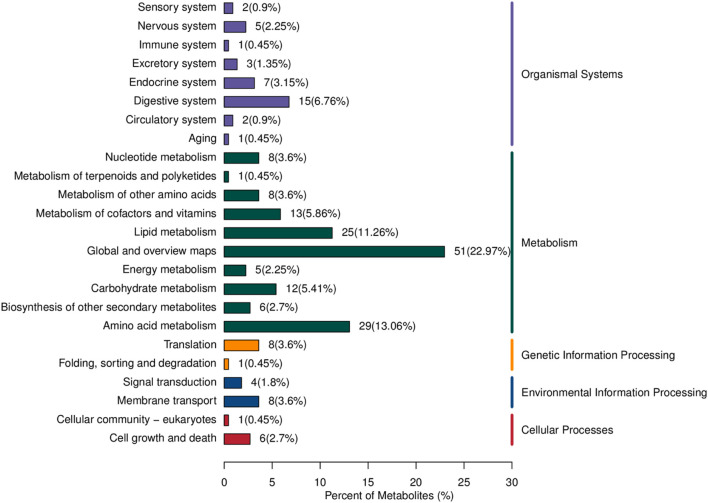
KEGG classification of different metabolites between PCA group and control group.

The differential metabolites between PCA group and control group were significantly enriched in various signaling pathways, as shown in the top 20 pathways listed in Table 6 and Figure 4. Key enriched pathways included ferroptosis, glycine, serine, and threonine metabolism, carbon metabolism, aminoacyl-tRNA biosynthesis, mineral absorption, the pentose phosphate pathway, protein digestion and absorption, nitrogen metabolism, pyrimidine metabolism, alanine, aspartate, and glutamate metabolism, D-glutamine and D-glutamate metabolism, biosynthesis of amino acids, phenylalanine metabolism, biosynthesis of phenylalanine, tyrosine, and tryptophan, glyoxylate and dicarboxylate metabolism, and glycolysis/gluconeogenesis. Figure 5 illustrates the KEGG classification of differential metabolites between PCA group and control group, with the top three categories being global and overview maps (22.97%), amino acid metabolism (13.06%), and lipid metabolism (11.26%).

**TABLE 6:** Differential metabolite enrichment pathways between PCA and control groups (top 20).

Map ID	Map title	P value	x
map04216	Ferroptosis	0.007	5
map00260	Glycine, serine and threonine metabolism	0.030	6
map01200	Carbon metabolism	0.030	6
map00970	Aminoacyl-tRNA biosynthesis	0.035	8
map04978	Mineral absorption	0.040	5
map02010	ABC transporters	0.049	8
map00860	Porphyrin and chlorophyll metabolism	0.052	4
map00030	Pentose phosphate pathway	0.066	3
map04974	Protein digestion and absorption	0.069	7
map00910	Nitrogen metabolism	0.076	2
map00240	Pyrimidine metabolism	0.096	4
map00250	Alanine, aspartate and glutamate metabolism	0.121	5
map00471	D-Glutamine and D-glutamate metabolism	0.132	3
map05230	Central carbon metabolism in cancer	0.132	3
map01230	Biosynthesis of amino acids	0.175	10
map00360	Phenylalanine metabolism	0.223	6
map00400	Phenylalanine, tyrosine and tryptophan biosynthesis	0.224	4
map00630	Glyoxylate and dicarboxylate metabolism	0.224	4
map00010	Glycolysis/Gluconeogenesis	0.277	1
map00524	Neomycin, kanamycin and gentamicin biosynthesis	0.277	1

## 4 Discussion

### 4.1 The influencing factors of PCA

The findings of this study indicates that smoking is a significant risk factor for PCA, aligning with previous research. Tobacco plays a role in all stages of AS development and is a key modifiable factor contributing to cardiovascular and cerebrovascular diseases (Centner et al., 2020). Numerous studies have confirmed that tobacco induces AS through multiple mechanisms, primarily targeting nitric oxide. The interference reduces the protective effects of nitric oxide on blood vessels while promoting oxidative stress (Murray et al., 2022). Oxidative stress is the central mechanism for AS pathogenesis, impairing vascular endothelium, activating platelets, increasing inflammatory factors, inhibiting fibrinolytic activity, and oxidizing lipoproteins, stimulating leukocytes (Addissouky et al., 2024). Recent studies have also identified a strong association between smoking, increased monocyte tissue factor activity, and accelerated progression of carotid intima-media thickness. Monocyte tissue factor activity is critical in both AS development and thrombosis formation (Kang et al., 2021).

Certain tobacco components impair vascular endothelial function by elevating inflammatory cytokines or triggering oxidative stress responses. The oxidative stress mechanism involves increased reactive oxygen species (ROS) production, which reduces endothelial nitric oxide synthase (eNOS) activity and nitric oxide levels. Concurrently, eNOS uncoupling perpetuates ROS generation, promoting vascular smooth muscle cell proliferation and matrix metalloproteinase secretion, further exacerbating endothelial dysfunction (Centner et al., 2020). Nicotine and lipopolysaccharides in tobacco might upregulate receptors of endothelin-1, angiotensin II, thrombin, and angiotensin I, leading to arterial vasoconstriction and endothelial cell dysfunction (Kim et al., 2023). Harmful tobacco components upregulate HSP60 mRNA levels in endothelial cells, which are transported to the cell surface after mitochondrial release and bind to macrophages to mediate immune responses. Furthermore, studies have demonstrated that the expression of HSP60 in endothelial cells represents a key mechanism underlying the pathogenesis of AS (Wang et al., 2024).

This study also suggests a correlation between unhealthy dietary habits and PCA onset. Hyperlipidemia and hyperuricemia, linked to high-salt and high-fat diets, are confirmed risk factors of AS, consistent with this study’s findings. Elevated uric acid levels are closely associated with the occurrence and severity of coronary atherosclerosis in young individuals, and hypertension combined with hyperuricemia significantly increases carotid AS incidence (Wei et al., 2023; Ubhadi et al., 2023).

Hypertension is an independent risk factor for AS. Studies have shown that age and systolic blood pressure are the primary risk factors for carotid AS and plaque formation (Chrysant, 2023). Hypertensive patients exhibit significantly greater common carotid artery intima-media thickness (IMT) compared to non-hypertensive individuals. Long-term uncontrolled hypertension can cause vascular endothelial damage through mechanical stimulation and other mechanisms, leading to lipid infiltration and deposition within arterial walls. This process further promotes the aggregation, adhesion, migration, and transformation of mononuclear macrophages into foam cells in the intima layer, significantly exacerbating AS progression and increasing the risk of cardiovascular and cerebrovascular events.

Some studies suggest that type 2 diabetes, a chronic inflammatory metabolic disease, is associated with pancreatic and adipose tissue inflammation, which may cause insulin resistance and impaired beta-cell function, ultimately leading to type 2 diabetes (Takeda et al., 2020). Defects in insulin secretion or action can result in abnormal glucose and lipid metabolism (Poznyak et al., 2020), a major complication of type 2 diabetes. The primary pathophysiological process involves AS formation, driven by chronic local inflammatory reactions mediated by cytokines produced by vascular endothelial cells, smooth muscle cells, macrophages, and lymphocytes on the vessel wall, along with lipid and cholesterol accumulation (Jebari-Benslaiman et al., 2022). Chronic immune inflammation in diabetic patients disrupts endocrine function and vasoactive factor release, elevating systemic levels of inflammatory markers. This leads to platelet and lipid deposition on vessel walls, macrophage aggregation, lumen narrowing, and changes in vascular permeability (Yu and Cheng, 2020).

### 4.2 The relationship between metabolic abnormalities and PCA

The findings of this study revealed significant abnormalities in lipid, glucose, and purine metabolism among patients with PCA. Specifically, TG and LDL-C levels were markedly elevated in PCA patients compared to the control group, while HDL-C, APOA, and the APOA1/APOB ratio were significantly reduced. These results suggest a strong association between PCA and lipid metabolism disorders. Previous research has established that lipid metabolism disorders are an independent risk factor for AS, playing a critical role in the initiation and progression of AS plaque formation and contributing to chronic inflammatory responses. Vascular endothelial cell dysfunction, a key driver of AS, is exacerbated by lipid metabolism disturbances, which disrupt the balance between endothelial cell proliferation and apoptosis. Clinical studies have demonstrated that elevated levels of TC and TG significantly impact AS plaque stability, as measured through biochemical assays and pulse wave analysis (Poznyak et al., 2023). Furthermore, high TG levels increase arterial stiffness, and concurrent the elevations of TC and TG levels synergistically promote plaque formation.

Lipoproteins in plasma are associated with specific apolipoproteins that serve distinct functions. For instance, ApoB is a primary component of LDL-C, while ApoA1 is the main constituent of HDL-C (Sniderman et al., 2019). ApoB levels directly correlate with LDL-C concentrations and are closely linked to AS progression and the onset of coronary atherosclerotic heart disease. Conversely, ApoA1 exhibits cholesterol-reversing, antithrombotic, antioxidant, and anti-atherogenic properties (Busnelli et al., 2021). Consequently, the ApoB/ApoA1 ratio serves as a dynamic indicator of the balance between AS promotion and inhibition. Current diagnostic and monitoring tools for AS include several lipid metabolism markers, among which LDL-C, HDL-C, ApoB, and ApoA1 have been strongly associated with AS diseases (Yaseen et al., 2021). The evidence suggests that the ApoB/ApoA1 ratio may provide a more accurate assessment of cardiovascular disease risks (Zhang et al., 2025).

Additionally, this study observed significantly higher serum UA levels in PCA patients compared to controls. UA, the end product of purine metabolism, activates oxidative stress pathways, leading to endothelial dysfunction. Elevated UA levels independently predict AS development and are associated with increased cardiovascular disease incidence and mortality across diverse populations (Dehlin et al., 2020; KimurabY and Kono, 2021). UA is also linked to metabolic abnormalities such as dyslipidemia, obesity, and diabetes (Yazdi et al., 2022). Experimental studies have demonstrated that UA reduces nitric oxide bioavailability in endothelial cells and induces insulin resistance, both *in vitro* and *in vivo*, thereby promoting AS (Bahadoran et al., 2022). However, the precise biological mechanisms that elevated UA increases cardiovascular risk remain unclear. Current evidence suggests that hyperuricemia is associated with lipid metabolism abnormalities, oxidative stress, hyperglycemia, and endoplasmic reticulum stress, all of which contribute to arterial damage (Lee et al., 2021; Gherghina et al., 2022; Piani et al., 2021; Li et al., 2022).

### 4.3 Metabolomics characteristics of PCA

Metabolomics serves as a powerful tool for disease diagnosis, pathogenesis research, and prevention, primarily through the analysis of metabolites closely associated with pathological conditions. Metabolomics technology enables the identification of specific biomarkers that can aid in disease diagnosis and support clinical decision-making. In this study, a non-targeted metabolomics approach utilizing LC-MS/MS was employed to identify potential plasma biomarkers in patients with PCA. A total of 1,171 metabolites were identified, with 693 detected in positive ion mode and 478 in negative ion mode. The observed metabolic alterations and pathways were predominantly associated with lipid metabolism, amino acids and their derivatives, and energy metabolism.

Among the top 30 metabolites identified in the PCA group, 14 were upregulated, while 16 were downregulated. Lipid metabolites constituted a significant proportion of these, with six phosphatidylcholines (PC 14:0_16:0, PC 16:0_18:1, PC 16:1_16:1, PC 20:1_18:2, PC 0–17:0_14:0, and PC 0–18:0_22:6) exhibiting altered metabolic levels. Additionally, three lysophosphatidylcholines (LPC 0–16:0, LPC 0–18:2, and LPC 0–16:1) were downregulated, and two phosphatidylethanolamines (PE 0–16:0_20:4 and PE-Cer 12:1; 20/22:0) were upregulated. Furthermore, LPE 0–22:2 was downregulated, while LPI 20:4 was upregulated, and Cer 29:0; 20/12:0; 0 (FA 18:0) was downregulated. These findings suggest that abnormal lipid metabolites may serve as metabolism markers for PCA.

This study focuses on the clinical precursor stage of atherosclerosis. During this early phase, reduced levels of LPC may indicate either the initial failure of the body’s defense mechanisms or early signs of metabolic dysregulation. In contrast, elevated LPC levels reported in the literature are primarily observed during advanced or acute stages of the disease, likely reflecting explosive LPC release following plaque rupture and extensive cellular necrosis (Liu et al., 2020). These phenomena represent distinct temporal windows in the continuum of disease progression. Furthermore, studies have demonstrated that circulating HDL-associated PC and LPC can be processed by vascular wall-associated enzymes, leading to localized lipid redistribution (Gauster et al., 2005). This suggests that the circulating LPC pool and the vascular wall LPC pool, while dynamically interconnected, are subject to independent regulation. The systemic decrease in circulating LPC levels thus does not contradict the localized accumulation of inflammation-driven LPC at lesion sites; together, these processes constitute the complex metabolic landscape of atherosclerosis.

Lipids perform essential biological functions in living organisms, serving as energy reservoirs, structural components of cellular membranes, and signaling molecules (Smirnov, 2010). Consequently, lipid metabolism serves as a sensitive indicator of physiological and pathological states in organisms. Extensive researches have established significant associations between dysregulated lipid metabolism and various pathological conditions, including obesity, AS, diabetes mellitus, and coronary heart disease (Soehnlein and Libby, 2021). As fundamental constituents of lipoproteins, phospholipids play a pivotal role in lipid metabolism. Insufficient phospholipids metabolism can lead to cholesterol deposition on arterial walls, contributing to AS (Jiang, 2020). Lysophosphatidylcholine, a key marker positively correlated with cardiovascular diseases, regulates low-density lipoprotein metabolism and plays a significant role in vascular endothelial dysfunction and AS plaque formation (Koenen, 2019; Seong et al., 2015). Ceramide, an intermediate metabolite of sphingolipids, is vital in biosynthesis and may protect vascular endothelium during early AS formation. Plasma ceramide levels have been shown to predict cardiovascular events and mortality risks in patients with arteriosclerotic cardiovascular disease more effectively than traditional biomarkers (Mantovani and Dugo, 2020; Rni et al., 2020; Peterson et al., 2018).

This study further PCA disrupts cellular energy metabolism, as indicated by elevated concentrations of phosphopyruvate, acetylcarnitine, and butyric acid. Acetylcarnitine plays a critical role in fatty acid metabolism and energy production by facilitating the transfer of fatty acids from the cytoplasm into the mitochondria, thereby promoting fat degradation and subsequent metabolic energy generation. During the final step of glycolysis, phosphoenolpyruvate is catalyzed by pyruvate kinase, transferring its phosphate group to ADP to produce ATP and pyruvate, releasing a significant amount of energy. Glycolysis serves as the primary energy metabolism pathway for endothelial cells under normal physiological conditions, supplying approximately 85% of their total ATP (De Bock et al., 2013). However, risk factors such as abnormal shear stress in the arterial walls of AS-prone regions can induce endothelial cell apoptosis, compromising the integrity of the endothelial barrier. Under pathological conditions, endothelial cells become activated, and glycolytic metabolism is significantly upregulated (Tricot et al., 2000), aligning with the findings of this study.

Additionally, the study identified abnormal amino acid metabolism, particularly involving N-acetylneuraminic acid, N-lactosyl-phenylalanine, tyrosine, and tryptophan, as a key feature of PCA. Notably, the metabolism of N-acetylneuraminic acid, a core structure of sialic acid, was found to be upregulated. Research indicates that sialic acid deposited in AS plaques can enhance collagen-induced platelet aggregation, adenosine triphosphate secretion, and platelet adhesion to fixed collagen. This effect is primarily mediated through sialic acid’s influence on the collagen-binding integrin α2β1 (Wen et al., 1999).

In summary, this study utilized non-targeted metabolomics approach to identify differential metabolites and enriched metabolic pathways in PCA. Despite its contributions, this study has several limitations. For instance, the sample size was limited, and the research methodology was relatively simplistic. Increasing the sample size and employing a multi-omics integrated analysis approach could enhance the accuracy and reliability of the findings. Future studies should prioritize expanding the sample size and incorporating multi-omics methods to validate and refine these results. Furthermore, large-scale, multi-center studies are necessary to confirm the findings of this research. A more comprehensive understanding of PCA-related differential metabolic markers and pathways can be achieved through targeted metabolomics validation combined with multi-omics analysis. Such an approach would provide valuable insights for the prevention and treatment of AS.

## Data Availability

The raw data supporting the conclusions of this article will be made available by the authors, without undue reservation.

## References

[B1] Addissouky T. A. El T.El S. I. Ali M. M. A. Wang Y. El Baz A. Elarabany N. (2024). Oxidative stress and inflammation: elucidating mechanisms of smoking-attributable pathology for therapeutic targeting. Bull. Natl. Res. Cent. 48, 16. 10.1186/s42269-024-01174-6

[B2] Agha R. A. Mathew G. Rashid R. Kerwan A. Al-Jabir A. Sohrabi C. (2025). Revised strengthening the reporting of cohort, cross-sectional and case-control studies in surgery (STROCSS) guideline: an update for the age of artificial intelligence. Premier J. Sci. 10, 100081. 10.70389/PJS.100081

[B3] Bahadoran Z. Mirmiran P. Kashfi K. Ghasemi A. (2022). Hyperuricemia-induced endothelial insulin resistance: the nitric oxide connection. Pflugers Arch. 474, 83–98. 10.1007/s00424-021-02606-2 34313822

[B4] Busnelli M. Manzini S. Chiara M. Colombo A. Fontana F. Oleari R. (2021). Aortic gene expression profiles show how ApoA-I levels modulate inflammation, lysosomal activity, and sphingolipid metabolism in murine atherosclerosis. Arterioscler. Thromb. Vasc. Biol. 41, 651–667. 10.1161/ATVBAHA.120.315669 33327742 PMC7837693

[B5] Caughey M. C. Qiao Y. Windham B. G. Gottesman R. F. Mosley T. H. Wasserman B. A. (2018). Carotid intima-media thickness and silent brain infarctions in a biracial cohort: the atherosclerosis risk in communities (ARIC) study. Am. J. hypertenion 10, 869–875. 10.1093/ajh/hpy022 29425278 PMC6049000

[B6] Centner A. M. Bhide P. G. Salazar G. (2020). Nicotine in senescence and atherosclerosis. Cells 9, 9041035. 10.3390/cells9041035 32331221 PMC7226537

[B7] Chrysant S. G. (2023). Association of hyperuricemia with cardiovascular diseases: current evidence. Hosp. Pract. 51, 54–63. 10.1080/21548331.2023.2173413 36730938

[B8] De Bock K. Georgiadou M. Schoors S. Kuchnio A. Wong B. W. Cantelmo A. R. (2013). Role of PFKFB3-driven glycolysis in vessel sprouting. Cell 154, 651–663. 10.1016/j.cell.2013.06.037 23911327

[B9] Dehlin M. Jacobsson L. Roddy E. (2020). Global epidemiology of gout: prevalence, incidence, treatment patterns and risk factors. Nat. Rev. Rheumatol. 16, 380–390. 10.1038/s41584-020-0441-1 32541923

[B10] Gauster M. Rechberger G. Sovic A. Hörl G. Steyrer E. Sattler W. (2005). Endothelial lipase releases saturated and unsaturated fatty acids of high density lipoprotein phosphatidylcholine. J. Lipid Res. 46, 1517–1525. 10.1194/jlr.M500054-JLR200 15834125

[B11] Gherghina M. E. Peride I. Tiglis M. Neagu T. P. Niculae A. Checherita I. A. (2022). Uric acid and oxidative stress-relationship with cardiovascular, metabolic, and renal impairment. Int. J. Mol. Sci. 23, 3188. 10.3390/ijms23063188 35328614 PMC8949471

[B12] Gianaros P. J. Rasero J. DuPont C. M. Kraynak T. E. Gross J. J. McRae K. (2022). Multivariate brain activity while viewing and reappraising affective scenes does not predict the multiyear progression of preclinical atherosclerosis in otherwise healthy midlife adults. Affect. Sci. 3, 406–424. 10.1007/s42761-021-00098-y 36046001 PMC9382946

[B13] Global C. R. C. Magnussen C. Ojeda F. M. Leong D. P. Alegre-Diaz J. Amouyel P. (2023). Global effect of modifiable risk factors on cardiovascular disease and mortality. N. Engl. J. Med. 389, 1273–1285. 10.1056/NEJMoa2206916 37632466 PMC10589462

[B14] Gulinuer D. Nuerguli M. (2023). The association between atherogenic index of plasma and all-cause mortality and cardiovascular disease-specific mortality in hypertension patients: a retrospective cohort study of NHANES. BMC Cardiovasc. Disord. 23, 452–464. 10.1186/s12872-023-03451-0 37697281 PMC10496369

[B15] Jebari-Benslaiman S. Galicia-García U. Larrea-Sebal A. Olaetxea J. R. Alloza I. Vandenbroeck K. (2022). Pathophysiology of atherosclerosis. Int. J. Mol. Sci. 23, 3346. 10.3390/ijms23063346 35328769 PMC8954705

[B16] Jiang X. C. (2020). Impact of phospholipid transfer protein in lipid metabolism and cardiovascular diseases. Adv. Exp. Med. Biol. 1276, 1–13. 10.1007/978-981-15-6082-8_1 32705590 PMC8025695

[B17] Kang H. Li X. Xiong K. Song Z. Tian J. Wen Y. (2021). The entry and egress of mono-cytes in atherosclerosis:a biochemical and biomechanicaldriven process. Cardiovasc Ther. 2021, 6642927. 10.1155/2021/6642927 34345249 PMC8282391

[B18] Kawai K. Finn A. V. Virmani R. Subclinical Atherosclerosis Collaborative (2024). Subclinical Atherosclerosis Collaborative. Subclinical atherosclerosis: part 1: what is it? Can it be defined at the histological level? Arterioscler. Thromb. Vasc. Biol. 44, 12–23. 10.1161/ATVBAHA.123.319932 38150517

[B19] Kim C. Y. Lee C. M. Lee S. Yoo J. E. Lee H. Park H. E. (2023). The Association of smoking status and clustering of obesity and depression on the risk of early-onset cardiovascular disease in young adults: a nationwide cohort Study. Korean Circ. J. 53, 17–30. 10.4070/kcj.2022.0179 36479644 PMC9834560

[B20] KimurabY T. D. Kono H. (2021). Uric acid in inflammation and the pathogenesis of atherosclerosis. Int. J. Mol. Sci. 22, 1–19. 10.3390/ijms222212394 PMC8624633 34830282

[B21] Koenen R. R. (2019). Lysophosphatidylcholine in platelet microvesicles: the grease for cardiovascular disease. Thrombosis Haemostasis 119, 1202–1204. 10.1055/s-0039-1693024 31266081

[B22] Lee T. S. Lu T. M. Chen C. H. Guo B. C. Hsu C. P. (2021). Hyperuricemia induces endothelial dysfunction and accelerates atherosclerosis by disturbing the asymmetric dimethylarginine/dimethylarginine dimethyl aminotransferase 2 pathway. Redox Biol. 46, 102108–102109. 10.1016/j.redox.2021.102108 34438260 PMC8390558

[B23] Li X. Liu M. Sun R. Zeng Y. Chen S. Zhang P. (2016). Atherosclerotic coronary artery disease: the accuracy of measures to diagnose preclinical atherosclerosis. Exp. Ther. Med. 12, 2899–2902. 10.3892/etm.2016.3710 27882093 PMC5103722

[B24] Li W. Wang Y. Ouyang S. R. Liu R. Zhang Y. (2022). Association between serum uric acid level and carotid atherosclerosis and metabolic syndrome in patients with type 2 diabetes mellitus. Front. Endocrinol. 13, 890305–890892. 10.3389/fendo.2022.890305 35769075 PMC9234212

[B25] Liu P. Zhu W. Chen C. Yan B. Zhu L. Chen X. (2020). The mechanisms of lysophosphatidylcholine in the development of diseases. Life Sci. 247, 117443. 10.1016/j.lfs.2020.117443 32084434

[B26] Mantovani A. Dugo C. (2020). Ceramides and risk of major adverse cardiovascular events: a meta-analysis of longitudinal studies. J. Clin. Lipidol. 14, 176–185. 10.1016/j.jacl.2020.01.005 32067904

[B27] Mfeukeu-Kuate L. Walinjom E. E. Mbede M. Walinjom J. N. Hamadou B. Jingi A. M. (2022). Prevalence and correlates of vascular plaques and high intima thickness in a group of patients with high cardiovascular risk in Cameroon. Pan Afr. Med. J. 41, 80. 10.11604/pamj.2022.41.80.31944 35382056 PMC8956831

[B28] Murray K. O. Berryman-Maciel M. Darvish S. Coppock M. E. You Z. Chonchol M. (2022). Mitochondrial-targeted antioxidant supplementation for improving age-related vascular dysfunction in humans: a study protocol. Front. Physiol. 13, 980783. 10.3389/fphys.2022.980783 36187760 PMC9520456

[B29] Peterson L. R. Xanthakis V. Duncan M. S. Gross S. Friedrich N. Völzke H. (2018). Ceramide remodeling and risk of cardiovascular events and mortality. J. Am. Heart Assoc. 7, e007931. 10.1161/JAHA.117.007931 29728014 PMC6015315

[B30] Piani F. Cicero A. F. G. Borghi C. (2021). Uric acid and hypertension: prognostic role and guide for treatment. J. Clin. Med. 10, 448. 10.3390/jcm10030448 33498870 PMC7865830

[B31] Poznyak A. Grechko A. V. Poggio P. Myasoedova V. A. Alfieri V. Orekhov A. N. (2020). The diabetes mellitus–atherosclerosis connection: the role of lipid and glucose metabolism and chronic inflammation. Int. J. Mol. Sci. 21, 1835. 10.3390/ijms21051835 32155866 PMC7084712

[B32] Poznyak A. V. Kashirskikh D. A. Postnov A. Y. Popov M. A. Sukhorukov V. N. Orekhov A. N. (2023). Sialic acid as the potential link between lipid metabolism and inflammation in the pathogenesis of atherosclerosis. Braz J. Med. Biol. Res. 56, e12972. 10.1590/1414-431X2023e12972 38088673 PMC10712282

[B33] Rni K. Jauhiainen M. Kovanen P. T. (2020). Why and how increased plasma ceramides predict future cardiovascular events? Atherosclerosis 314, 71–73. 10.1016/j.atherosclerosis.2020.09.030 33121744

[B34] SCORE2 working group and ESC Cardiovascular risk collaboration (2021). SCORE2 risk prediction algorithms: new models to estimate 10-year risk of cardiovascular disease in Europe. Eur. Heart J. 42, 2439–2454. 10.1093/eurheartj/ehab309 34120177 PMC8248998

[B35] Seong H. H. Hyun H. J. So R. L. Lee K. H. Woo J. S. Kim J. B. (2015). Impact of lysophosphatidylcholine on survival and function of UEA-1+ acLDL+ endothelial progenitor cells in patients with coronary artery disease. Heart Vessels 30, 115–125. 10.1007/s00380-014-0473-z 24510253

[B36] Smirnov A. N. (2010). Lipid signaling in the atherogenesis context. Biochem. Mosc. 75, 793–810. 10.1134/s0006297910070011 20673203

[B37] Sniderman A. D. Thanassoulis G. Glavinovic T. Navar A. M. Pencina M. Catapano A. (2019). Apolipoprotein B particles and cardiovascular disease: a narrative review. JAMA Cardiol. 4 (4), 1287–1295. 10.1001/jamacardio.2019.3780 31642874 PMC7369156

[B38] Soehnlein O. Libby P. (2021). Targeting inflammation in atherosclerosis from experimental insights to the clinic. Nat. Rev. Drug Discov. 20, 589–610. 10.1038/s41573-021-00198-1 33976384 PMC8112476

[B39] Sun D. X. Du Y. H. Li R. F. Zhang Y. (2025). Metabolomics for early-stage lung adenocarcinoma: diagnostic biomarker screening. Front. Oncol. 11, 1535525–13. 10.3389/fonc.2025.1535525 40134589 PMC11932905

[B40] Takeda Y. Matoba K. Sekiguchi K. Nagai Y. Yokota T. Utsunomiya K. (2020). Endothelial dysfunction in diabetes. Biomedicines 8, 182. 10.3390/biomedicines8070182 32610588 PMC7400447

[B41] Touboul P. J. Hennerici M. G. Meairs S. Adams H. Amarenco P. Bornstein N. (2012). Mannheim carotid intima-media thickness and plaque consensus (2004-2006-2011): an update on behalf of the advisory board of the 3rd, 4th, and 5th watching the risk symposia, at the 13th, 15th, and 20th European Stroke Conferences, Mannheim, Germany, 2004, Brussels, Belgium, 2006, and Hamburg, Germany, 2011. Cerebrovasc. Dis. Basel Switz. 34, 290–296. 10.1159/000343145 23128470 PMC3760791

[B42] Tricot O. Mallat Z. Heymes C. Belmin J. Lesèche G. Tedgui A. (2000). Relation between endothelial cell apoptosis and blood flow direction in human atherosclerotic plaques. Circulation 101, 2450–2453. 10.1161/01.cir.101.21.2450 10831515

[B43] Ubhadiya T. J. Dubey N. Sojitra M. H. Shah K. Joshi S. Gandhi S. K. (2023). Exploring the effects of elevated serum uric acid levels on hypertension: a scoping review of hyperuricemia. Cureus 15, e43361. 10.7759/cureus.43361 37701002 PMC10494276

[B44] Wang S. X. Chen Y. Q. Zhou D. Y. Zhang J. Guo G. (2024). Pathogenic autoimmunity in atherosclerosis evolves from HSP60-Reactive CD4 + T cells. J. Cardiovasc Transl. Res. 17, 1172–1180. 10.1007/s12265-024-10516-8 38767798

[B45] Wei X. Zhang M. Huang S. Lan X. Zheng J. Luo H. (2023). Hyperuricemia:a key contributor to endothelial dysfunction in cardiovascular diseases. FASEB J. 37, e23012. 10.1096/fj.202300393R 37272854

[B46] Wen F. Q. Jabbar A. A. Patel D. A. Kazarian T. Valentino L. A. (1999). Atherosclerotic aortic gangliosides enhance integrin-mediated platelet adhesion to collagen. Arterioscler. Thromb. Vasc. Biol. 19, 519–524. 10.1161/01.atv.19.3.519 10073952

[B47] Xiao J. Q. Gu C. Q. He S. Zhu D. Huang Y. Zhou Q. (2021). Widely targeted metabolomics analysis reveals new biomarkers and mechanistic insights on chestnut (Castanea mollissima Bl.) calcification process. Food Res. Int. 141, 110128. 10.1016/j.foodres.2021.110128 33641995

[B48] Xue X. M. Tian S. L. Chen R. Han X. Wang J. Zhao X. (2022). Clarifying the mechanisms of the light - induced color formation of Apple peel under dark conditions through metabolomics and transcriptomic analyses. Front. Plant Sci. 13, 946115. 10.3389/fpls.2022.946115 35968118 PMC9366354

[B49] Yaseen R. El-leboudy M. El-deeb H. (2021). The relation between Apo B/Apo A-1 ratio and the severity of coronary artery disease in patients with acute coronary syndrome. Eur. Heart J. 73, 24. 10.1186/s43044-021-00150-z 33725226 PMC7966664

[B50] Yazdi F. Baghaei M. H. Baniasad A. Naghibzadeh-Tahami A. Najafipour H. Gozashti M. H. (2022). Investigating the relationship between serum uric acid to high-density lipoprotein ratio and metabolic syndrome. Endocrinol. Diabetes Metab. 5, e00311. 10.1002/edm2.311 34705333 PMC8754234

[B51] Yu W. Cheng J. D. (2020). Uric acid and cardiovascular disease: an update from molecular mechanism to clinical perspective. Front. Pharmacol. 11, 582680–590. 10.3389/fphar.2020.582680 33304270 PMC7701250

[B52] Zhang J. Liu M. Y. Gao J. Tian X. Song Y. Zhang H. (2025). ApoB/ApoA-Ι is associated with major cardiovascular events and readmission risk of patients after percutaneous coronary intervention in one year. Sci. Rep. 15, 996. 10.1038/s41598-024-84092-x 39762288 PMC11704328

